# Predictors of adverse events after percutaneous pedicle screws fixation in patients with single-segment thoracolumbar burst fractures

**DOI:** 10.1186/s12891-022-05122-1

**Published:** 2022-02-22

**Authors:** Shengtao Dong, Zongyuan Li, Zhi-ri Tang, Yuanyuan Zheng, Hua Yang, Qiuming Zeng

**Affiliations:** 1 Department of Spine Surgery, the Second Affiliated Hospital of Dalian Medical University, Dalian, 116023 China; 2 Department of Orthopedics, Mianyang Central Hospital, Mianyang, 621000 China; 3 School of Physics and Technology, Wuhan University, Wuhan, 430072 China; 4 Department of Oncology, the Second Affiliated Hospital of Dalian Medical University, Dalian, 116023 China; 5 Department of Otolaryngology, the Second Affiliated Hospital of Dalian Medical University, Dalian, 116023 China; 6 Department of Orthopedics, University-Town Hospital of Chongqing Medical University, Chongqing, 401331 China

**Keywords:** Thoracolumbar burst fracture, Percutaneous pedicle screws fixation, Risk factors, Support vector machine

## Abstract

**Background:**

Percutaneous pedicle screw fixation (PPSF) is the primary approach for single-segment thoracolumbar burst fractures (TLBF). The healing angle at the thoracolumbar junction is one of the most significant criteria for evaluating the efficacy of PPSF. Therefore, the purpose of this study was to analyze the predictors associated with the poor postoperative alignment of the thoracolumbar region from routine variables using a support vector machine (SVM) model.

**Methods:**

We retrospectively analyzed patients with TLBF operated at our academic institute between March 1, 2014 and December 31, 2019. Stepwise logistic regression analysis was performed to assess potential statistical differences between all clinical and radiological variables and the adverse events. Based on multivariate logistic results, a series of independent risk factors were fed into the SVM model. Meanwhile, the feature importance of radiologic outcome for each parameter was explored. The predictive performance of the SVM classifier was evaluated using the area under the receiver operating characteristic curve (AUC), accuracy (ACC) and confusion matrices with 10-fold cross-validation, respectively.

**Results:**

In the recruited 150 TLBFs, unfavorable radiological outcomes were observed in 53 patients (35.33%). The relationship between osteoporosis (*p* = 0.036), preoperative Cobb angle (*p* = 0.001), immediate postoperative Cobb angle (*p* = 0.029), surgically corrected Cobb angle (*p* = 0.001), intervertebral disc injury (Score 2 *p* = 0.001, Score 3 *p* = 0.001), interpedicular distance (IPD) (*p* = 0.001), vertebral body compression rate (VBCR) (*p* = 0.010) and adverse events was confirmed by univariate regression. Thereafter, independent risk factors including preoperative Cobb angle, the disc status and IPD and independent protective factors surgical correction angle were identified by multivariable logistic regression. The established SVM classifier demonstrated favorable predictive performance with the best AUC = 0.93, average AUC = 0.88, and average ACC = 0.87. The variables associated with radiological outcomes, in order of correlation strength, were intervertebral disc injury (42%), surgically corrected Cobb angle (25%), preoperative Cobb angle (18%), and IPD (15%). The confusion matrix reveals the classification results of the discriminant analysis.

**Conclusions:**

Critical radiographic indicators and surgical purposes were confirmed to be associated with an unfavorable radiographic outcome of TLBF. This SVM model demonstrated good predictive ability for endpoints in terms of adverse events in patients after PPSF surgery.

## Introduction

The thoracolumbar junction (T10-L2) is the most frequent site of spinal fracture due to that it forms a transition region between the relatively fixed thoracic spine above and a relatively mobile lumbar spine below, resulting in a concentration of stress on the thoracolumbar vertebrae [1, 2]. Approximately 160,000 thoracolumbar fractures occur annually in North America, with 20% of these consisting of thoracolumbar burst fractures (TLBF) [3]. The fracture line involves both the anterior and middle columns of the spine [4], with potential complications including pain, paralysis and deformity, placing a tremendous financial burden on these patients [5, 6].

The study by McAfee et al. first identified the contribution of fracture morphology to the evaluation of vertebral stability and the determination of treatment options [7]. Based on this, Magerl et al. proposed a comprehensive classification system designed to suggest fracture severity and instability through a continuum of grades and indirectly to obtain the state of the nervous system [8]. It is important to note that due to the complexity of the Magerl classification and the neurological injuries obtained by extrapolation, it has not fully acquired the confidence of clinical surgeons. Furthermore, the Thoracolumbar Injury Classification System (TLICS) emphasizes the neurological status, integrity of the posterior ligamentous complex (PLC) and morphology of thoracolumbar fractures and assigns separate values to them to facilitate differentiation between the surgical and conservative treatment groups [9]. This system has a total score of 10, and patients with a score of less than 3 are considered to be the target population for conservative treatment. However, given the controversy over the reproducibility and feasibility of magnetic resonance imaging (MRI) assessment criteria, further clarification of the role of PLC integrity in thoracolumbar spine fractures is still necessary [10–12]. The new AO TL injury classification system introduced by Vaccaro et al. provides spine surgeons with a convenient and reproducible classification system that includes morphological classification of fractures, neurological status grading, and specific fracture modifiers [13].

Percutaneous pedicle screws fixation (PPSF) has become a routine treatment option for single-segment TLBF because of their ability to achieve fixation of the fractured vertebrae and correction of the deformity based on reduced damage to paraspinal tissues, reduced intraoperative blood loss and early painless mobilization [14–17]. However, compared to posterior open pedicle screw fixation, PPSF is inherently flawed in terms of ensuring osseointegration and endograft safety due to the failure to add bone grafts and transverse connectors [18, 19].

In latest years, machine learning (ML) models have been widely applied in the field of orthopedics and have shown good performance [20–22]. Support vector machine (SVM), proposed by VAPNIK two decades ago, is a classification method for both linear and nonlinear data. The input vector is mapped to a high-dimensional feature space by some pre-selected nonlinear mapping, and the optimal classification hyperplane is constructed in this space. Finally, the support variables are exactly the nearest sample points.

The published articles have discussed several routine radiological indicators with respect to T-L junction Cobb angle, interpedicular distance, etc. However, reliable predictors and predictive models to assess long-term radiological outcomes after PPSF are lacking. This work aimed to analyze the predictors of adverse events after PPSF with TLBF by using the SVM classifier.

## Methods

### Study design and participants

We retrospectively analyzed patients with TLBFs operated at our academic institute between March 1, 2014, and December 31, 2019. The inclusion criteria: (1) a single-level traumatic TLBF (T11-L2), (2) thoracolumbar fracture classified as A3 or A4 according to the new AO Spine classification reported by Vaccaro [13], (3) age 18 to 65 years, (4) no other significant injury, (5) no history of tumor and not pathologic thoracolumbar fracture, (6) the interval between injury and surgery is less than 15 days, (7) posterior stabilization procedure only with percutaneous pedicle screws and rods, (8) postoperative follow-up for at least 1 year. Exclusion criteria were a history of spinal surgery or obsolete spinal fractures, multi-segmental vertebral fractures, pregnancy, and refusal of surgery. Preoperatively, complete radiological parameters and physical findings were obtained for all patients, and all surgeons in the department were involved in making the surgical decision and agreed to perform PPSF.

Clinical characters included gender, age, BMI, diabetes, hypertension, smoking, visual analog scale (VAS, 0–10), and mechanisms of injury (traffic accidents, falls from a height, same level falls, and sport). Radiological assessment of the sagittal Cobb angle, the intervertebral disc injury, vertebral body compression rate (VBCR), percentage of anterior height compression (PAHC), canal compromise (CC), interpedicular distance (IPD), and osteoporosis were recorded and compared. All patients were followed up for at least 12 months.

We defined the adverse event group as patients with a sagittal Cobb angle greater than 20 degrees in the thoracolumbar junction at the 1-year follow-up, or patients with a Cobb angle greater than 10 degrees from the immediate postoperative status to the final follow-up, or with significant instrumentation failures, such as nonunion of the fracture or screw loosening as well as rod or screws rupture [23].

### Operative technique

All operations are performed by senior spine surgeons. After induction of general anesthesia, the patient was placed on a radiolucent operating table in the prone position, with pads under the pelvis and thorax to obtain postural reduction. Prior to incision of the skin, C-arm fluoroscopy was performed in the anterior-posterior and lateral planes to ensure that the pedicle could be sufficiently observed. Meanwhile, the six access portals (approximately 2 cm in length) for internal fixation implantation are marked compared to the preoperative radiographs. A guide wire is used to penetrate the fascia and then a series of consecutive dilators are used to dilate the fascia and separate the underlying paraspinal muscles. Under fluoroscopic guidance, a vertebral guide hole is made using a tracked awl and a pedicle probe. A guide wire is placed into the selected pedicle and a pedicle screw with an expander is then slid in. The screws are fixed to the fractured vertebral body and the adjacent vertebral body above and below [16, 24]. Fluoroscopic C-arm guidance was used throughout the pedicle screw implantation. The angle of the instrumentation was adjusted intraoperatively to determine satisfactory fixation. Any intraoperative bleeding was controlled by bipolar forceps, closing the fascial incision and suturing the skin. Only pedicle screws and nail rods were used, and no graft fusion was performed. We confirmed and stored the last C-arm fluoroscopic image with a satisfactory correction angle before suturing the incision, and the immediate postoperative Cobb angle was measured from these plain radiographs.

Postoperative patients are allowed to roll in bed, sit and ambulate with the support of a molded brace, and are discharged when they feel significant pain relief and are comfortable with normal life.

### Radiological parameter measurement

All measurements are full spine radiographs and as much as possible within the patient’s tolerable pain range is required to obtain the radiographs in a standing position, except for the immediate postoperative Cobb angle.

**Fig. 1:**
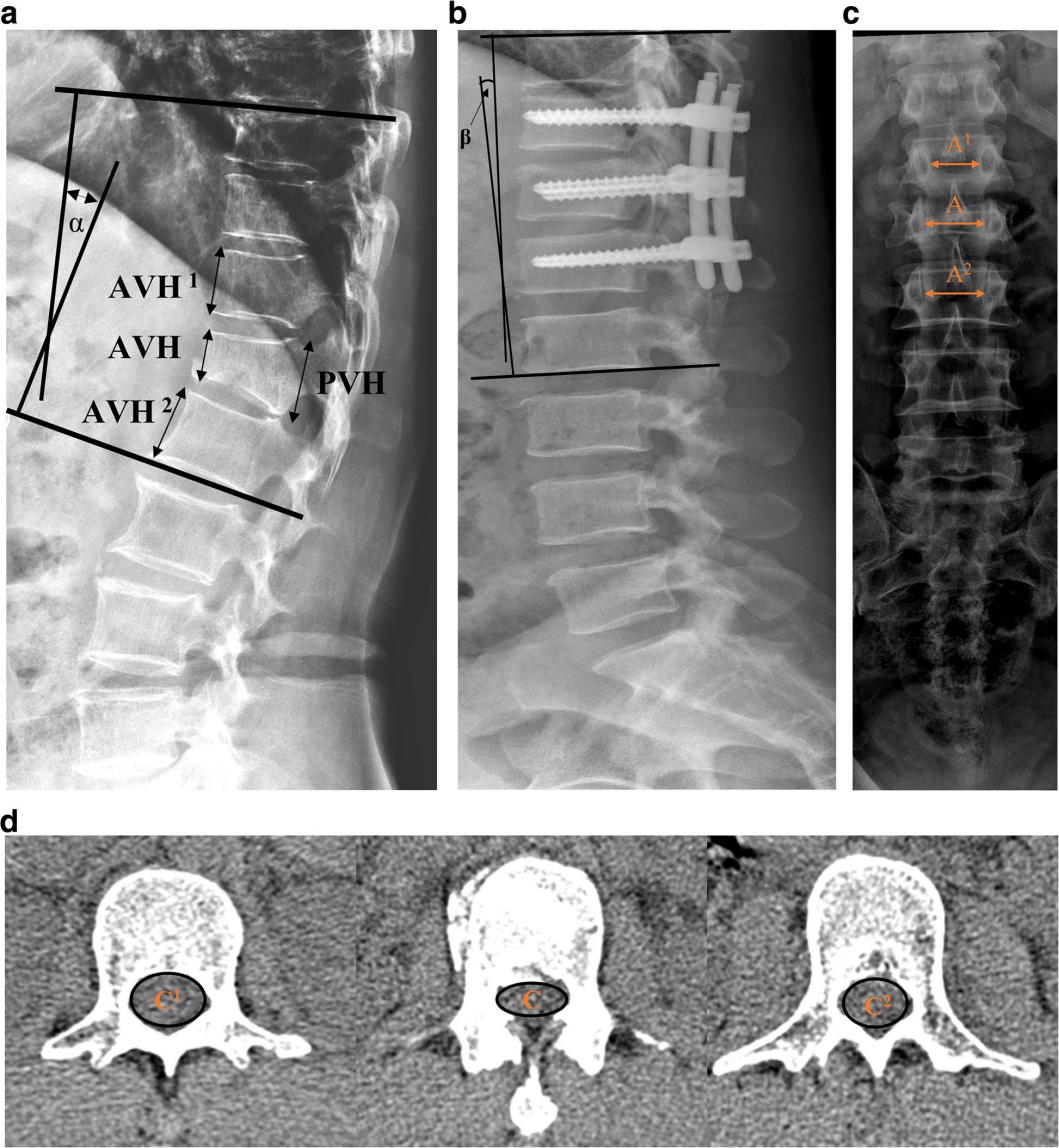
**a**, **b** the preoperative Cobb angle and surgically corrected Cobb angle are α and β, respectively. AVH^1^: Anterior vertebral height of a vertebra above the fracture, AVH^2^: Anterior vertebral height of a vertebra below the fracture, PVH: posterior vertebral height of fractured vertebra. **c** the interpedicular distance. **d** the canal compromise, the canal area of the injured and adjacent uninjured segments

The Cobb angle was the angle at the thoracolumbar junction that contains the fractured vertebrae and is located between the upper endplate of T10 and the lower endplate of L2. Surgically corrected Cobb angle was a difference that calculated from the patient’s first post-injury imaging and the immediate postoperative imaging. MRI examinations determined the status of the intervertebral discs adjacent to the fractured vertebrae (1.5-T Magnetom Avanto, Siemens). Compared to the adjacent discs, morphological changes were evaluated using a 0–3 scoring system [25]. Vertebral body compression rate (VBCR) is a ratio of anterior to the posterior vertebral body height of the fractured segment (VBCR = AVH/PVH × 100%). Percentage of anterior height compression (PAHC) is a ratio of the anterior vertebral height between the fractured segment and the adjacent segments (AVH/[(AVH^1^ + AVH^2^)/2] × 100%). Canal compromise (CC) is a ratio of the canal area of the injured segment to the average of two adjacent uninjured levels that are considered as canal compromise. The intervertebral distance (IPD) was calculated by taking the measured width between the pedicles of the fractured vertebrae and the similar values of the adjacent upper and lower vertebrae according to the following formula: IPD = [2A - (A^1^ + A^2^)/ (A^1^ + A^2^)] × 100%. A T-score ≤ − 2.5 measured by dual X-ray absorptiometry (DEXA) was diagnosed as osteoporosis (Fig. 1).

To obtain the reliable results in the present study, all radiological measurements were performed by two experienced spine surgeons independently. We determined the final values from the average of the measurements taken by both.

### Support vector machine

Artificial intelligence (AI) is already making a splash in healthcare by inspiring a paradigm shift in medical data processing. AI aims to create mimics that resemble human cognition, where one of the classic supervised machine learning approaches for structured data is the SVM model. The basic assumption of SVM is that subjects can be divided into two groups by defined classification boundary. The goal of training is to find the best classification hyperplane in the space so that the obtained hyperplane is as consistent as possible with the results. Meanwhile, the determination of the SVM model parameters is a convex optimization problem, so the solution is always globally optimal. In this study, based on the results of 10-fold cross-validation, the performance of the SVM model was evaluated by the area under the receiver operating characteristic curve (AUC), accuracy (ACC) and confusion matrix, respectively. Moreover, the feature importance between variables and clinical outcomes was also analyzed and evaluated. Python 3.8 and the Scikit-learn 0.21.2 package were used to build our SVM classifier.

### Statistical analysis

Continuous variables are presented as means ± standard deviations (SD), and categorical variables are presented as relative frequencies and percentages. For normally distributed continuous variables, Student’s *t* tests were applied. Otherwise, the Mann Whitney test was adopted. For categorical variables, the chi-square test was used. In addition, we applied logistic regression analysis to identify radiological parameters and clinical characteristics associated with the adverse events. The variables that proved to be statistically different in the univariate regression analysis were regarded as the initial risk factors. They were then analyzed by multivariate regression to detect the independent risk factors. Furthermore, the selected variables were put into the SVM classifier. *p* < 0.05 was considered statistically significant. All statistical analysis was performed with SPSS version 22.0 (IBM SPSS, Armonk, New York).

## Results

### Included patients

A total of 150 consecutive patients were recruited, and 53 patients (35.33%) fulfilled the inclusion criteria for the adverse events. The overall age of the patients was 45.59 ± 11.80, 44.88 ± 12.44 in the non-adverse event group and 46.89 ± 10.40 in the adverse event group. Males predominated in both groups. No statistical differences in BMI, hypertension, diabetes, smoking, or VAS were identified between the adverse event and non-adverse event groups. We found significantly more patients with osteoporosis in the adverse event group (30.19%) compared to the non-adverse event group (15.46%, *p* = 0.033). Both groups indicated that the primary mechanism of injury was a traffic accident (60.38% vs. 56.64%), and T12 level is the most commonly injured segment (Table 1). Radiological variables showed a general correlation with adverse events, except for PAHC and CC. Notably, in the adverse event group, the preoperative Cobb angle (18.34 ± 4.03), immediate postoperative Cobb angle (8.11 ± 5.02) and IPD were greater, the surgically corrected angle and VBCR was smaller (5.78 ± 3.00), and more patients were diagnosed with severe disc impairment (Score 2: 28.30% vs 15.46%, Score 3: 49.06% vs 11.34%). (Table 2).

**Table 1:** Comparison of the clinical characteristics

Variable	Total	AE group	Non-AE group	*p*
Number of patients	150	53	97	
Age (years)	45.59 ± 11.80	46.89 ± 10.40	44.88 ± 12.44	0.322
Sex (%)				0.802
Female	70 (46.67)	24 (45.28)	46 (47.42)	
Male	80 (53.33)	29 (54.72)	51 (52.58)	
BMI (kg/ m^2^)	24.44 ± 2.34	24.17 ± 1.71	24.59 ± 2.60	0.239
Hypertension (%)	26 (17.33)	10 (18.87)	16 (16.50)	0.714
Diabetes (%)	44 (29.33)	18 (33.96)	26 (26.80)	0.357
Smoking (%)	28 (18.67)	13 (24.53)	15 (15.46)	0.173
VAS	5.98 ± 1.21	5.98 ± 1.19	5.98 ± 1.22	0.993
Osteoporosis (%)	31 (20.67)	16 (30.19)	15 (15.46)	0.033
Cause of injury (%)				0.797
Traffic accidents	85 (56.67)	32 (60.38)	53 (54.64)	
Falls from a height	44 (29.33)	13 (24.53)	31 (31.96)	
Same level falls	14 (9.33)	5 (9.43)	9 (9.28)	
Sport	7 (4.67)	3 (5.66)	4 (4.12)	
Injury level (%)				0.772
T11	19 (12.67)	6 (11.32)	12 (12.37)	
T12	63 (42.00)	22 (41.51)	39 (40.21)	
L1	45 (30.00)	20 (37.74)	30 (30.93)	
L2	23 (15.33)	5 (9.43)	16 (16.49)	

*BMI* body mass index, *VAS* Visual analogue scores

**Table 2:** Comparison of the radiologic variables

Variable	Total	AE group	Non-AE group	*p*
Preop. Cobb angle (°)	16.63 ± 3.73	18.34 ± 4.03	15.69 ± 3.12	< 0.001
Postop. Cobb angle (Immediate, °)	6.73 ± 5.44	8.11 ± 5.02	5.98 ± 5.51	0.022
Postop. Cobb angle (1-year, °)	15.62 ± 6.16	23.10 ± 1.84	11.53 ± 3.07	< 0.001
Surgically corrected Cobb angle (°)	8.26 ± 3.67	5.78 ± 3.00	9.60 ± 3.27	< 0.001
Intervertebral disc injury (%)				< 0.001
Score 0	36 (24.00)	4 (7.55)	32 (32.99)	
Score 1	47 (31.33)	8 (15.09)	39 (40.21)	
Score 2	30 (20.00)	15 (28.30)	15 (15.46)	
Score 3	37 (24.67)	26 (49.06)	11 (11.34)	
IPD (%)	23.04 ± 1.82	24.06 ± 1.82	22.48 ± 1.56	< 0.001
VBCR (%)	68.00 ± 3.53	66.98 ± 3.27	68.55 ± 3.55	0.009
PAHC (%)	69.43 ± 2.94	68.83 ± 3.20	69.75 ± 2.73	0.065
CC (%)	10.17 ± 3.57	10.88 ± 3.45	9.78 ± 3.57	0.074

*Preop* preoperative, *Postop* postoperative, *IPD* interpedicular distance, *VBCR* vertebral body compression rate, *PAHC* percentage of anterior height compression, *CC* canal compromise. *Score 0* an intact, undamaged disc, *Score 1* a hypertonic edematous image of the disc was observed, *Score 2* a ruptured disc with intradiscal hemorrhage, *Score 3* the disc was recessed into the vertebral body, had an annular tear, or herniated out into the lamina

### Correlation of variable with clinical outcome

Univariate logistic regression initially screened out variables with statistical differences, including osteoporosis (*p* = 0.036), preoperative Cobb angle (*p* = 0.001), immediate postoperative Cobb angle (*p* = 0.029), surgically corrected Cobb angle (*p* = 0.001), intervertebral disc injury (Score 2 *p* = 0.001, Score 3 *p* = 0.001), IPD (*p* = 0.001), and VBCR (*p* = 0.010). Multivariable logistic regression confirmed significant associations between preoperative Cobb angle (*p* = 0.002), surgically corrected Cobb angle (*p* = 0.004), intervertebral disc injury (Score 3 *p* = 0.011) and IPD (*p* = 0.015) and the adverse events. Independent risk factors included preoperative Cobb angle, the disc status, and IPD, and independent protective factors were surgical correction angle. (Table 3).

**Table 3:** Relationship between variables and adverse events by univariate and multivariate logistic regression analysis

Variables	Univariable logistic regression analysis				Multivariable logistic regression analysis			
	OR	95% CI		*p*	OR	95% CI		*p*
		Lower	Upper			Lower	Upper	
Osteoporosis	2.364	1.057	5.333	0.036	2.330	0.730	7.766	0.157
Preop. Cobb angle	1.224	1.111	1.361	0.001	1.236	1.089	1.419	0.002
Postop. Cobb angle (Immediate)	1.073	1.009	1.147	0.029	1.060	0.958	1.174	0.265
Surgically corrected Cobb angle	0.718	0.635	0.803	0.001	0.787	0.661	0.922	0.004
Intervertebral disc injury								
Score 0	Ref	Ref	Ref	Ref	Ref	Ref	Ref	Ref
Score 1	1.641	0.471	6.608	0.451	1.551	0.337	7.911	0.579
Score 2	8.000	2.440	31.975	0.001	2.900	0.619	15.010	0.183
Score 3	18.909	5.883	75.763	0.001	6.941	1.654	33.912	0.011
IPD	1.708	1.378	2.174	0.001	1.411	1.078	1.891	0.015
VBCR	0.876	0.789	0.967	0.060				
PAHC	0.897	0.796	1.006	0.067				
CC	1.090	0.992	1.202	0.075				

*OR* odds ratio, *95% CI* 95% confidence interval.

### Support vector machine

**Fig. 2:**
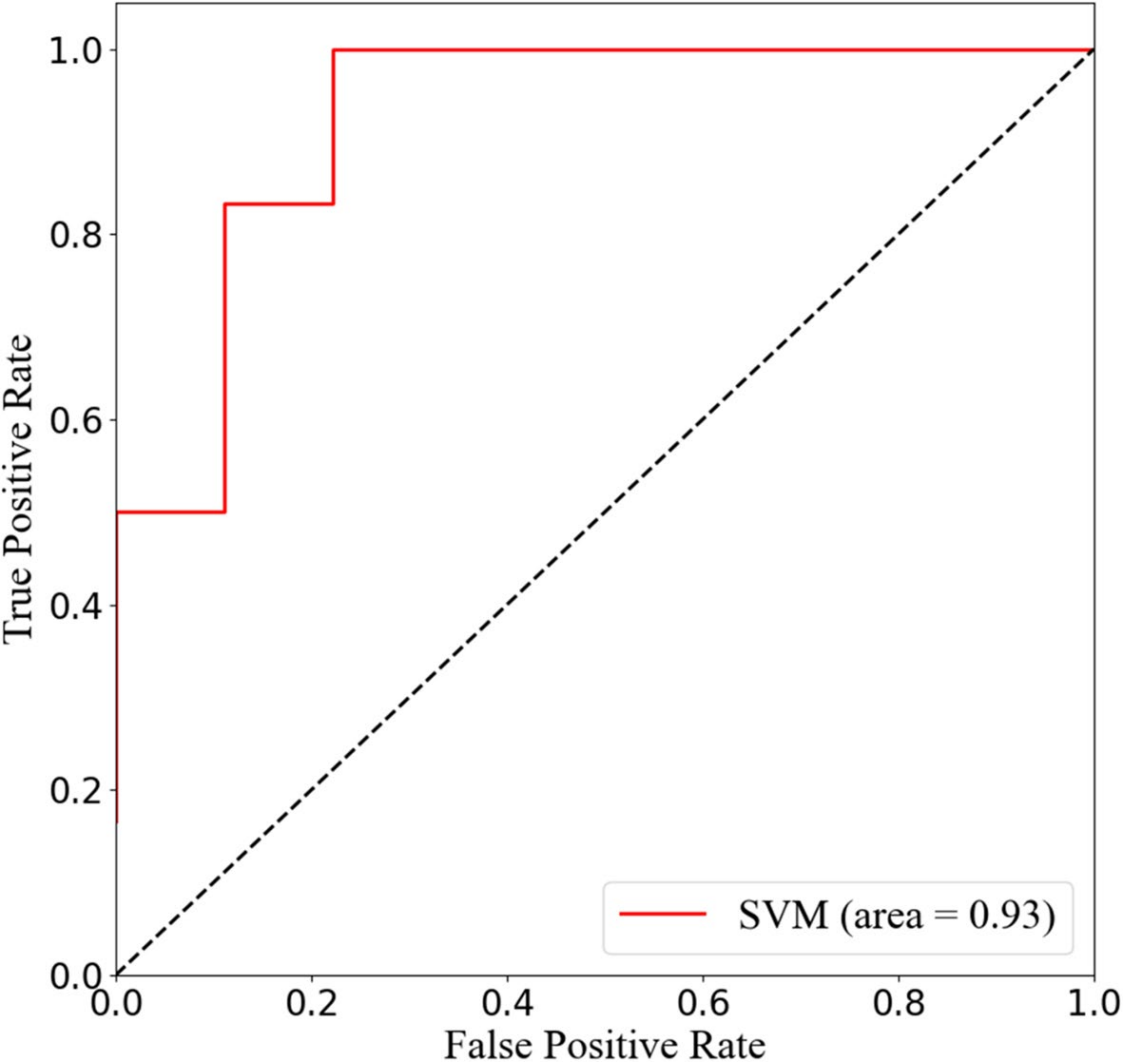
Receiver operation characteristic (ROC) curve analysis of SVM model with the maximum value 0.93

**Fig. 3:**
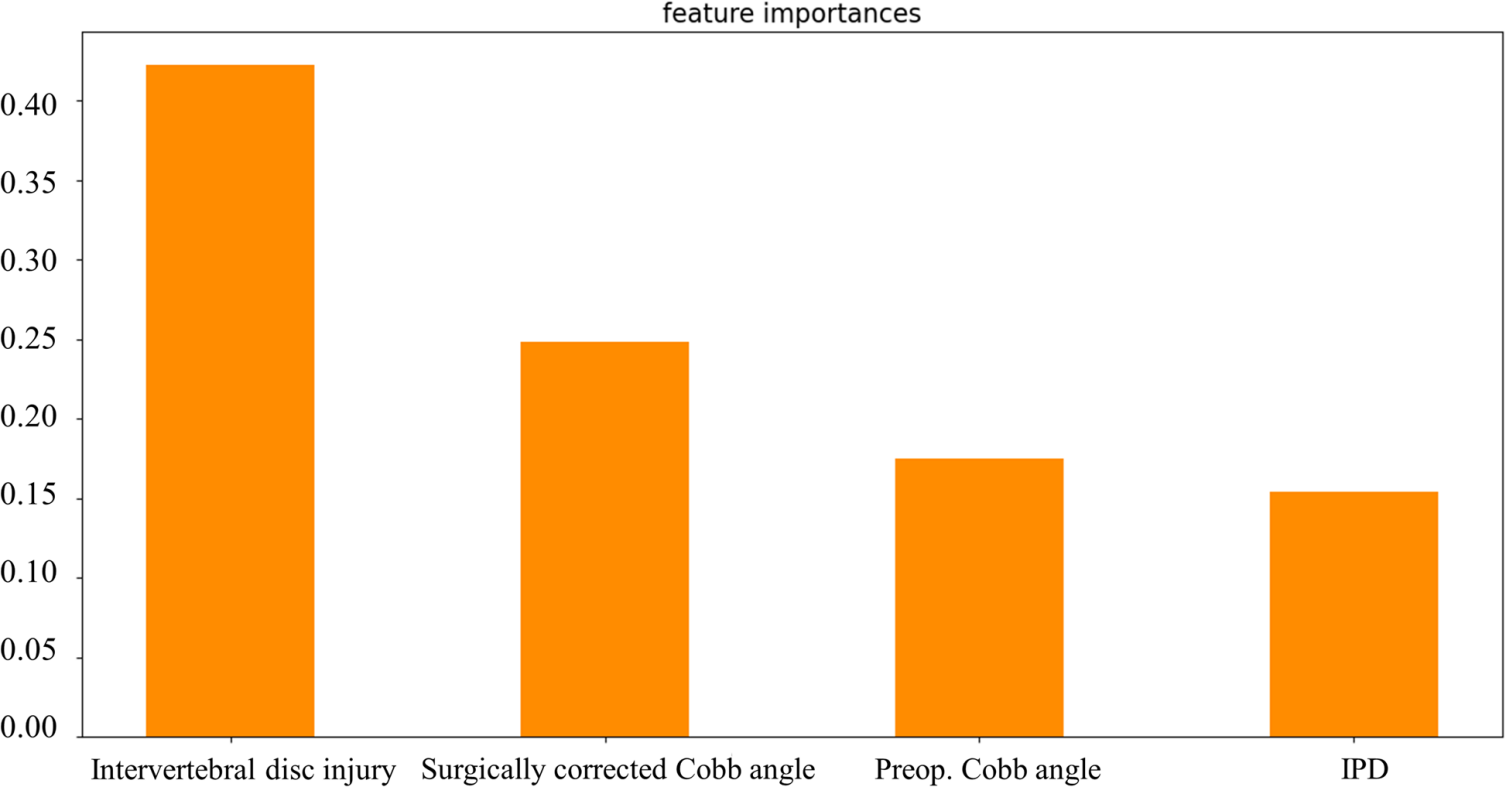
The feature importance analysis

**Fig. 4:**
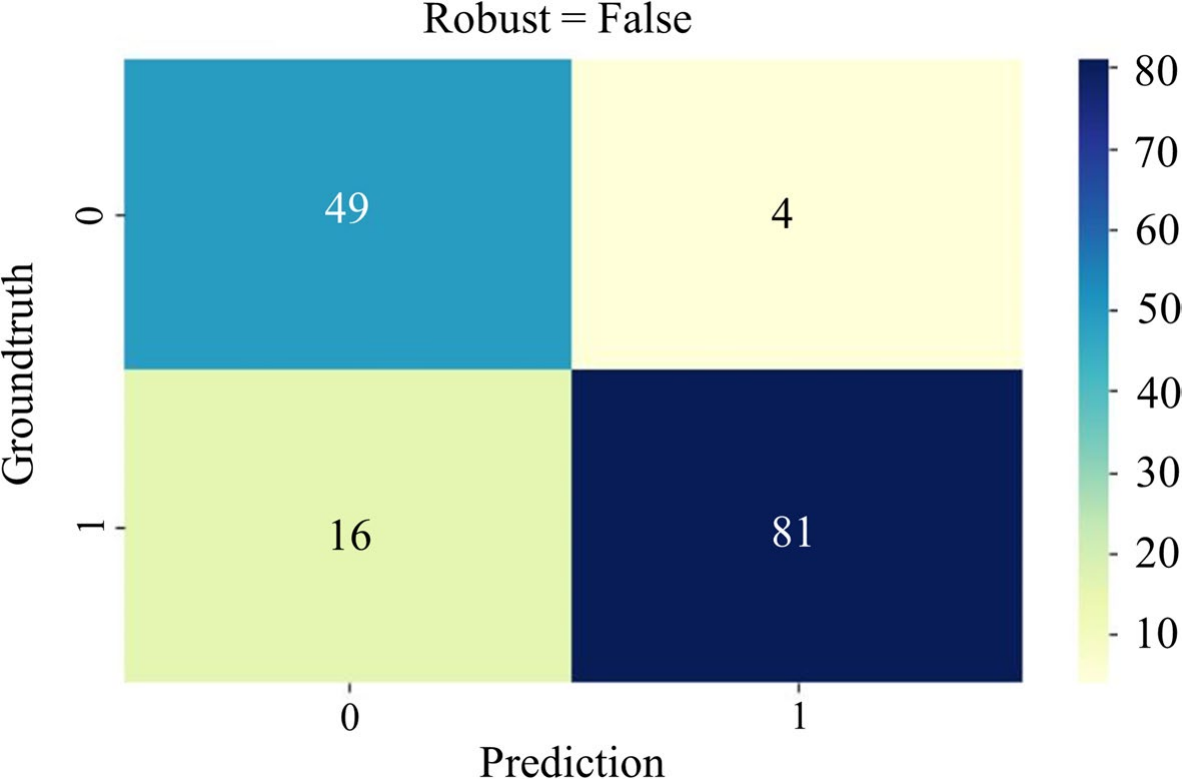
Confusion matrix of SVM model with good predictive ability

The variance results of multivariate logistic regression were incorporated into the ML model, including preoperative Cobb angle, surgically corrected Cobb angle, intervertebral disc injury, and IPD. Combined with the results of 10-fold cross-validation, the receiver operating characteristic (ROC) curves of the SVM model demonstrated favorable predictive ability with best AUC = 0.93 and average AUC = 0.88. The average ACC = 0.87, respectively (Fig. 2). The feature importance indicated that the disc status possessed the best predictive indication (42%), and the others were surgically corrected Cobb angle (25%), preoperative Cobb angle (18%), and IPD (15%) in that order, as shown in Fig. 3. The classification results of the discriminant analysis are further demonstrated by the confusion matrix (Fig. 4).

## Discussion

In this study, we determined the relationship between radiological characteristics and adverse events. Based on machine learning algorithms, a SVM model with excellent performance was established involving TLBF undergoing the PPSF technique. Intervertebral disc injury, Cobb angle and IPD have been confirmed to be independent risk factors for achieving good alignment in TLBF after this approach.

Compared to the adverse event group, fewer patients in the counterpart group were diagnosed with osteoporosis (30.19% vs. 15.46%). However, we did not find that osteoporosis significantly affected the prognosis of patients in a multivariate regression analysis. Previous reports have proposed the conclusion that osteoporosis is responsible for malunion of the vertebral body [26–28]. Kanno et al. highlighted the role that osteoporosis plays in vertebral fractures and that any factor that may elevate the risk of fracture is magnified for the thoracolumbar transitional region [19]. The debate over this conclusion continued, as Pesenti et al. argued that a distinction should be clearly made between fracture type (burst or compression) and age when considering the interaction between osteoporosis and thoracolumbar fractures [29]. In the present study, we excluded patients over 65 years of age. It is well known that age and the prevalence of osteoporosis are significantly associated, so this may explain the fact that our logistic regression results do not support the classification of osteoporosis as an independent risk factor.

Many classification systems for thoracolumbar fracture have attempted to assess and validate the influence of fractured vertebral body morphology on clinical outcomes. However, there was a need to highlight the association between traumatic disc lesions and patient radiological outcomes. A classifier of thoracolumbar traumatic disc status was reported by Sander et al. [25]. Furthermore, the disc adjacent to the fracture was found to be at risk of deterioration at one-year follow-up, with 50% of intact discs progressing to fibrous annulus rupture and/or disc filling the fracture gap [25, 30]. Xu et al. found that after short-segmental pedicle fixation of thoracolumbar fractures, the correction of kyphosis is lost under disc impairment during up to 8 years of follow-up [31]. In the present study, we likewise corroborate the critical position of disc status and hypothesize mechanisms. Due to the lack of blood perfusion, the regenerative capacity of the adult intervertebral disc is inadequate, making the upper or lower endplates of the fractured vertebrae susceptible to accelerated degeneration [25]. The cause of injury also provides insight into the interpretation of traumatic disc status. The majority of our recruited patients belonged to high-energy trauma (56.67% of car accident injuries, 29.33% of falls from height, 4.67% of sports), where the huge vertical loading force at the time of fracture breaks the vertebral endplates and adjacent discs, squeezing the discs into the vertebral body and causing pathological changes [2, 32]. However, without stabilization techniques for the disc space, patients with PPSF alone-only are at risk of exhibiting kyphotic malposition during follow-up due to traumatic disc lesions. As described, the nucleus pulposus that invades the fractured vertebral body causes collapse of the cancellous skeleton. However, posterior PPSF alone only improves the vertebral lordosis, the pathological changes between the broken nucleus pulposus, fibrous annulus and adjacent endplates were not corrected. Incomplete discs are usually fully distracted through ligamentotaxis, but load conduction is impaired. While satisfactory kyphosis was achieved, the spine stabilized solely posteriorly was forced to carry previous vertical loads due to the lack of anterior load sharing and exhibits axial load-related instrumentation failure at long-term follow-up. Given that the superior endplate is more severely injured in a burst fracture, redistribution and attachment of the nucleus pulposus to the endplate results in load dysregulation of the superior endplate and more significant narrowing of the superior adjacent disc is observed.

The Cobb angle was often referred to describe the grade of the kyphosis and has been shown to correlate significantly with the prognosis of TLBF [17, 23, 33–35]. In our study, larger preoperative (18.34 ± 4.03 vs. 15.69 ± 3.12) and smaller surgical correction (5.78 ± 3.00 vs. 9.60 ± 3.27) Cobb angles measured by thoracolumbar plain radiographs indicated these patients were at greater risk of deterioration to adverse events. Accordingly, there is sufficient evidence to believe that the kyphosis status at the moment of injury and the surgical operation are important references to optimize long-term outcomes. Among all the predictors assessing the severity of TLBF, spine surgeons have achieved consensus on the contribution of preoperative Cobb angle. However, the intervention of surgery on the Cobb angle still requires to be further elaborated.

Compared to conventional open approaches, previous studies have proposed the primary features of PPSF are less surgical trauma (reduced retraction extent, minimal destruction of paraspinal muscle tissue and blood vessels, shortened operative duration, decreased intraoperative bleeding, and low infection rate) [14, 15, 17] and shorter surgery-work intervals [16, 36, 37]. The postoperative sagittal Cobb angle and anterior vertebral body height achieved with this minimally invasive technique are comparable to the open counterpart [17]. In addition, patients undergoing PPSF can see an improvement in postoperative clinical outcomes, including VAS, length with hospitalization, and early mobilization [16, 38]. Because PPSF requires fluoroscopy throughout the process, we provide surgeons with critical surgical references to expeditiously achieve surgical goals and avoid excessive radiation exposure doses. A smaller preoperative Cobb angle and a larger surgically corrected cobb angle are suggestive factors for a long-term favorable radiological outcome. Distraction should be considered at the time of rod implantation to achieve bracing of the fractured segment and maintenance of the intervertebral space height, the deterioration of the latter causing increased load-bearing of the posterior column structures and disturbed zygapophysial joint correspondence, further accelerating disc degeneration and being the pathological basis for exacerbation and or recurrence of postoperative low back pain [15, 38]. Therefore, preoperative partial reduction and intraoperative distraction of the injured segment are both essential steps to improve the radiographic and clinical outcome of TLBF [16, 17, 36, 37].

The pedicle distance is one profound and vulnerable region, and its stability plays an important role in assessing the severity of TLBF. Measuring the internal trabecular structure of the vertebral body, Zhao et al. found that the bone cortex at the base of the pedicle was thinner [39]. In addition, axial trauma concentrates greater forces in the area, resulting in an increased distance between the pedicles of the fractured vertebrae [40]. The intervertebral foramen serves as a fundamental channel for vascular access to the vertebral body. Widened IPD might ripple through this structure, which eventually kyphotic malposition due to reduced blood supply [2]. Retropulsed bone fragments (RBFs) also exemplify the importance of assessing pedicle impairment to guide TLBF treatment [41, 42]. As mentioned previously, more retrograde bone fragments lead to more spinal canal compression and more severe neurological impairment due to widening IPD. On the one hand, displacement of the fracture fragments is a consensus cause of poor radiological outcomes [3]. In particular, spinal cord compression leading to neurological deficit is the most dreaded complication. It is necessary to add the measurement of IPD to the schedule for the diagnosis of TLBF, both to develop optimal treatment and to predict the long-term prognosis of fracture patients. In contrast, no relationship between CC and fractured vertebral alignment was observed in the present study. This may be because we included only mild patients classified as A3 or A4, and patients already had neurological deficits or were at high risk were not targeted.

Two patients presented with internal fixation failure, as evidenced by loosening and withdrawal of the screws, and both were diagnosed with osteoporosis. This finding emphasizes that rigid bone is the cornerstone for successful PPSF and is consistent with published studies [19, 37, 43–45]. Percutaneous instrumentation with cement augmentation helps to strengthen osteoporotic vertebrae and fill TLBF fractures with significant defects after deformity correction. In addition, PPSFs with short segments may loosen and break screws due to excessive overload forces. For TLBF with an intact posterior column, placement of pedicle screws above and below the fractured segment may improve segmental structure and biomechanical stability [16, 46].

Patients were classified according to radiological outcomes, with instrumentation failure and recurrence of kyphosis being the endpoint events of interest in this work. Although our study focused on identifying radiological predictors in patients, it was equally useful to facilitate clinical recovery. On the one hand, substantial studies have confirmed the association between T-L junction Cobb angle and functional outcome, including that patients with postoperative Cobb angles less than 10° report favorable functional outcomes and that poor functional outcomes are usually significantly associated with follow-up Cobb angles greater than 20° [23, 31, 47–51]. These evidences are the theoretical cornerstones of this study. We found that the mean postoperative Cobb angle was 5.98 (less than 10°) in the favorable group of patients and 23.1 (greater than 20°) in the poor group of patients at final follow-up, both consistent with previous findings. On the other hand, considering that patients’ tolerance to pain is not entirely consistent, one more standardized postoperative functional assessment system is necessary, including return to work status, daily exercise, etc.

The combination of multivariate logistic regression analysis and SVM machine learning algorithm was one of the attractive flashes of this study. The AUC and ACC of the prediction model were outstanding at 0.88 and 0.87, respectively, and the confusion matrix confirmed the replicability of the results. There were several limitations that need to be mentioned. First, we retrospectively collected data from our single-center of patients. The conclusions are limited by the sample size and research design. Second, the constructed prediction model lacks sufficient external validation. Third, a follow-up period of only one year may have missed potentially unfavorable patients. Therefore, a study with a follow-up period of 5 years is the focus of our future research. Finally, based on practice and a summary of the published literature, we considered the mentioned variables but may still overlook other important parameters.

## Conclusion

The proposed SVM model focused on several important radiological predictors in TL burst fractures. Intervertebral disc injury, preoperative Cobb angle, surgically corrected Cobb angle, and IPD were independent risk factors for the adverse events in patients with TLBF one year after PPSF. Therefore, adequate and accurate preoperative evaluation and purposeful intraoperative manipulation were issues that spine surgeons should think about. This finding helps to provide a basis for surgeons to develop an optimal treatment plan with the goal of favorable long-term postoperative vertebral anatomy.

## Data Availability

All data generated or analyzed during this study are included in this published article [and its supplementary information files].

## Abbreviations

PPSF: Percutaneous pedicle screw fixation
TLBF: Thoracolumbar burst fractures
AUC: Area under the receiver operating characteristic curve
ACC: Accuracy
TLICS: The Thoracolumbar Injury Classification System
MRI: Magnetic resonance imaging
VBCR: Vertebral body compression rate
PAHC: Percentage of anterior height compression
CC: Canal compromise
IPD: Interpedicular distance
ML: Machine learning
SVM: Support vector machine
